# Fracture resistance of simulated immature teeth treated with a regenerative endodontic protocol

**DOI:** 10.1080/23337931.2019.1570822

**Published:** 2019-01-31

**Authors:** Mohamed Raouf W. Ali, Manal Mustafa, Asgeir Bårdsen, Athanasia Bletsa

**Affiliations:** a Department of Clinical Dentistry Faculty of Medicine, University of Bergen, Bergen, Norway;; b Oral Health Centre of Expertise in Western Norway, Bergen, Norway

**Keywords:** Fracture resistance, MTA, Biodentine, TotalFill, bovine teeth

## Abstract

This study aims to evaluate fracture resistance of simulated immature teeth after treatment with regenerative endodontic procedure (REP) using tricalcium silicate cements (TSCs) as cervical plugs. Bovine incisors were sectioned to standard crown/root ratio. Pulp tissue was removed and canals were enlarged to a standardized diameter. Teeth were then treated with a REP protocol consisting of NaOCl and EDTA irrigation, intracanal medication with triple-antibiotic paste for 14 days followed by a TSC cervical seal and composite restoration. Teeth were divided into groups according to the material used; Mineral-Trioxide-Aggregate (MTA), Biodentine, TotalFill. Teeth filled with guttapercha (GP) and intact teeth served as controls. All teeth subjected to an increasing compressive force (rate of 0.05 mm/s at a 45° angle to the long axis of the tooth) until fracture. All treated teeth exhibited significantly lower resistance to fracture compared to the intact teeth but no difference was found between the TSC groups (Kruskal-Wallis, Dunn’s multiple comparison, *p* < .05). TSCs applied at the cervical area of simulated immature teeth treated with REP did not reinforce fracture resistance.

## Introduction

Endodontic treatment of non-vital immature permanent teeth presents quite a challenge in dental clinics due to wide open apices and thin dentinal walls. A relatively high incidence of cervical root fracture (>60%) has been reported in such teeth teeth after a long-term intra-canal treatment with calcium hydroxide (CH) in order to achieve a hard-tissue barrier at the apical area (apexification) [1,2]. These fractures may occur with minor impacts or spontaneously over time [1,3]. In the latest years, tricalcium silicate cements (TSC) have been widely used as endodontic repair materials and dentin substitutes [4]. The use of TSC materials to achieve a root-end closure at the apical area of necrotic immature teeth (direct apexification) has replaced the traditional treatment with CH. However, with this method the dentinal walls remain thin, and the risk of fracture is still present [5,6].

Regenerative endodontic procedures (REP) have been advocated as an alternative treatment modality to apexification for immature permanent teeth with necrotic pulp [7]. Regenerative endodontics have been defined as ‘‘biologically based procedures designed to replace damaged structures, including dentin and root structures, as well as cells of the pulp-dentin complex’’ [8] with the optimal goal to regenerate functional pulpal tissue and subsequently further root development. Although there is no consensus regarding the clinical regenerative protocols [9,10] the common step in all suggested ones is cervical sealing with a TSC barrier. This biocompatible cervical plug provides a bacterial-tight seal and acts as pulp space barrier [11,12].

The fact that non-vital immature teeth, due to fragile root, are more prone to fracture represents a substantial clinical problem. The risk of fracture of endodontically treated immature teeth relates to the degree of root development, with lower degree of development associated with higher fracture risk [1]. REPs aim at inducing further root development and eventually strengthening the tooth. However, even with REPs, the cervical area does not develop further. Furthermore, placement of TSCs at this exact area may mechanically affect the susceptibility of treated immature teeth to fracture. Little is known about the immediate effect of TSCs on the fracture resistance of immature teeth treated with REPs. The aim of this *in vitro* study was to investigate the fracture resistance of simulated immature teeth treated with REP and sealed at the cervical area with three different TSC materials; White MTA ANGELUS® (MTA), Biodentine™ Septodont (Biodentine), and TotalFill^®^ BC RRM™ Putty (TotalFill).

The null hypotheses tested:

H_0_, there is no difference in fracture resistance between intact immature bovine teeth and immature bovine teeth treated with different TSCs as coronal seal during REP.

## Material and methods

### Bovine teeth preparation

**Figure 1.**
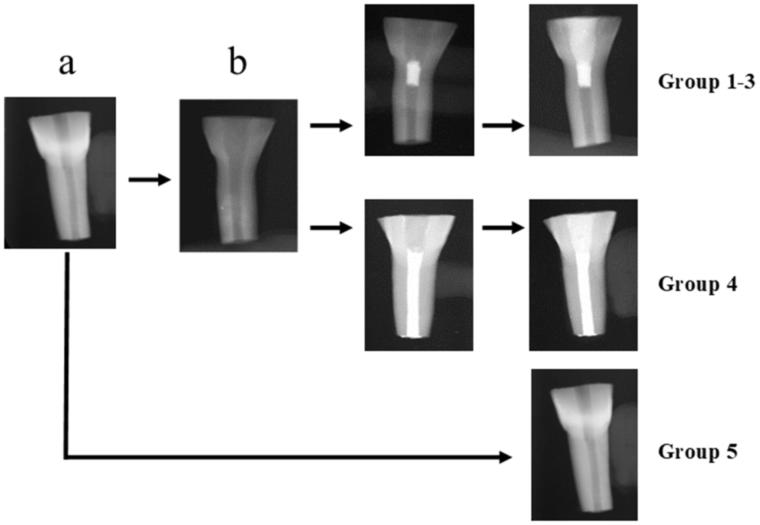
Flow-chart showing teeth preparation. Bovine incisors were first sectioned to standard a certain crown/root ratio (a). Canals were thereafter prepared to simulate immature teeth (b). These teeth were divided to groups (1–4) according to the filling material used (1: MTA, 2: Biodentine, 3: TotalFill, 4: Gutta-percha). Some sectioned teeth, remained unprepared and served as controls (group 5).

Bovine mandibular incisor teeth were extracted, cleaned and stored in 1% Benzalkonium Chloride [13]. Teeth were examined thoroughly and teeth with visible cracks/fractures were discarded. Intact teeth were then prepared according to a standard protocol in order to simulate immature teeth. Briefly, they were sectioned with a water cooled low speed diamond bur, coronally 10 mm above the cemento-enamel junction (CEJ) and apically 15 mm below the CEJ. The root canal was thereafter instrumented and widened with a size 6 peeso reamer so that an ISO size #120 file could pass completely unhindered throughout the canal. In that way, the internal canal diameter and the remaining dentin thickness were standardized close to 2 mm [5,6,14–16] (Figure 1).

Controls (intact teeth) were sectioned according to the standardized crown/root ratio but the canal was not prepared (Figure 1).

Dentine thickness and canal diameter at the cervical area of all teeth was measured with buccolingual and mesiodistal radiographs using the DIGORA Optime UV system (Unident, Falkenberg, Sweden) and the measurements were averaged.

### Tricalcium silicate cement materials

The TSC shown in Table 1 were mixed according to the manufacturer’s instructions and used in the regenerative endodontic procedure and throughout this study.

**Table 1.** Summary of the Tricalcium Silicate Cements (TSC) cements used in the study.

White MTA-Angelus^®^ (Angelus, Londrina, PR, Brazil)	Biodentine™ (Septodont, Saint-Maurdes Fosses, France)	TotalFill^®^ BC RRM™ PUTTY (FKG Dentaire, La-Chaux-de-Fonds, Switzerland)
**Powder:** Tricalcium silicate, dicalcium silicate, tricalcium aluminate, calcium oxide, iron tetracalcium aluminate, bismuth oxide; **Liquid:** distilled water **Mixing ratio:** 1 scoop of powder to 1 drop of liquid	**Powder:** Tricalcium and dicalcium silicate, calcium carbonate and zirconium oxide; **Liquid:** water, calcium chloride and modified polycarboxylate. **Mixing ratio:** 5 drops of liquid into powder capsule	**Ready-made paste:** Calcium silicates, zirconium oxide, tantalum pentoxide, calcium phosphate monobasic and filler agents

### Regenerative endodontic procedure

Simulated immature teeth were by a random procedure allocated into the 4 groups; MTA (*n* = 11) (group 1), Biodentine (*n* = 10) (group 2), TotalFill (*n* = 10) (group 3), Gutta Percha (GP) (*n* = 10) (group 4). In addition, untreated teeth served as controls (intact teeth, *n* = 10) (group 5) (Figure 1). Intact teeth were stored in a wet flower arrangement foam in a 37 °C and 100% humidity incubator until testing. All teeth in groups 1–4 were treated with the protocol followed at the dental clinics of the University of Bergen: irrigation with 10 ml Dakin’s solution (0.5% buffered sodium hypochlorite) followed by 5 ml of 17% ethylenediamine tetraacetic acid (EDTA) and 5 ml sterile water. The canals were then dried with paper points and filled with a triple antibiotic paste consisted of equal volumes of 500 mg Metronidazole, 500 mg Ciprofloxacin and 500 mg Amoxicillin mixed with sterile water in a slurry paste placed with a lentulo spiral. The access cavities were then sealed with Cavit^®^ temporary filling material and the roots inserted into a wet flower-arrangement foam. The teeth were stored in incubator (37 °C and 100% humidity) for 10 days [6]. The Cavit^®^ was then removed and the triple antibiotic paste was washed out with the same irrigation protocol as above. Teeth in groups 1–3 were sealed with a cervical plug of TSC. Teeth in group 4 were obturated with gutta-percha using lateral condensation technique and sealer (AH Plus^®^ DENTSPLY, Germany) and avoiding overfilling by applying finger pressure at the apex. Those teeth served as negative controls (Figure 1). Buccolingual and mesiodistal radiographs were taken to measure the material plug length (measurements were averaged as stated previously) and to confirm the uniformity of the gutta-percha obturation using the DIGORA Optime UV system. A wet cotton pellet and Cavit^®^ temporary filling material was placed at the access cavity and the teeth were stored overnight in the incubator to allow the TSC to set. After complete setting of the TSC material, a composite filling (3 M ESPE Filtek™ Supreme XTE) was placed as the coronal seal using a 4^th^ generation bonding system involving the use of 38% phosphoric acid (TOP DENT etch gel 2.5 ml, DAB DENTAL, Sweden) followed by a primer application (Optibond™ FL) and Adhesive (Optibond™ FL). Group 4 teeth were filled with composite immediately after filling with GP and stored in a wet flower arrangement foam in the incubator (37 °C and 100% humidity) until testing (Figure 1). Buccolingual radiographs were again taken to confirm the integrity of the composite fillings using the DIGORA Optime UV (Unident, Falkenberg, Sweden).

### Fracture resistance testing

All teeth were dipped into molten wax leaving a 0.2–0.3 mm thick layer of wax covering the root (2 mm below the CEJ to the root apex) [14]. Thereafter, the roots were embedded in acrylic resin cylinders (Heraeus, MELIODENT^®^ Rapid Repair, Denture acrylic self-curing, Kulzer, Germany) that were prepared using polyvinyl chloride cylinder molds measuring 20 mm in diameter and 17 mm high [15]. As soon as polymerization of the acrylic resin started, the teeth were removed from the resin, and the wax was cleaned from the root surfaces using a curette. The cleaned root surfaces were then coated with a thin layer of polyvinylsiloxane impression material (Affinis®, Coltene/Whaledent AG, Altstatten, Switzerland) to simulate the periodontal ligament (PDL) [5,14,17–19] and then re-embedded into the acrylic resin block. The acrylic block with the prepared teeth was mounted onto an MTS^®^ Hydraulic test System and subjected to an increasing compressive force at a test rate of 0.05 mm/s while being positioned at 45° angle to the long axis of the tooth until fracture occurred [5,6,14–16,20]. Peak load at fracture was recorded in Newton (N).

### Statistical analysis

For statistical analysis GraphPad Prism5 (GraphPad Software, La Jolla, CA, USA) was used. D’Agostino-Pearson omnibus normality test validated the distribution of the data. For normally distributed data one-way analysis of variance (ANOVA) with Bonferroni’s *post hoc* test was used. When normality test was not passed, Kruskal-Wallis with Dunn’s *post hoc* test was used for the comparison between the groups or Mann-Whitney test for comparison between two groups (e.g. prepared vs intact teeth). All tests were performed at a level of significance *α* = 0.05. Results are presented as mean ± SEM, (*p < .05; **p < .01; ***p < .001).

## Results

### Simulated immature teeth

**Figure 2.**
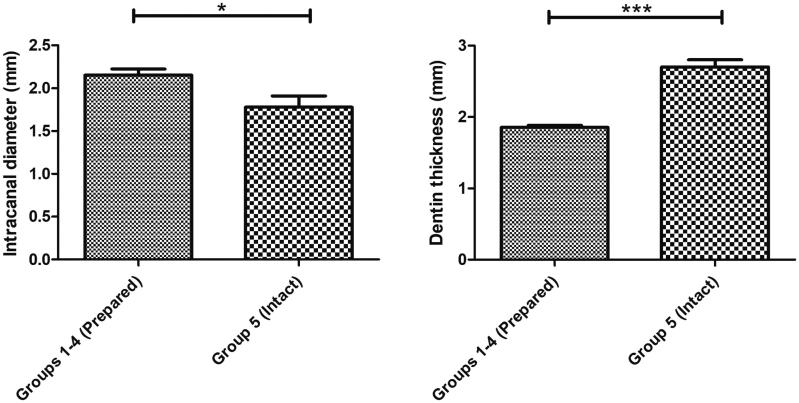
The simulated immature teeth (groups 1–4, *n* = 41) had a statistically significant larger canal diameter (2,153 ± 0,07 mm) and lower dentin thickness measured at the CEJ (1,857 ± 0,027 mm) compared to the intact teeth (1,780 ± 0,13 mm and 2,704 ± 0,098 mm, respectively) (group 5, *n* = 10); Results are presented as mean ± SEM, Mann-Whitney test, *p < 0.05; ***p < 0.001.

In the interest of experimental standardization, there were no differences between the simulated immature teeth (groups 1–4) in terms of crown/root ratio, intra-canal diameter at CEJ and dentin thickness (Table 2). Moreover, there were no differences between the TSC groups (1–3) in terms of the TSC cervical plug length (MTA: 3.9 ± 0.193 mm; Biodentine: 4.015 ± 0.1228 mm and TotalFill: 3.481 ± 0.125 mm) (Table 2). All prepared teeth (groups 1–4) had a significantly higher canal diameter and lower dentin thickness measured at the CEJ compared to the intact teeth (group 5) (Figure 2 and Table 2, *p* < .05).

**Table 2.** Dimensions (mean ± SEM) of the bovine teeth used in the study.

Group (*n* = number of teeth)	Crown/Root ratio	Intra-canal Diameter at CEJ (mm)	Dentine Thickness at CEJ (mm)	Cervical Plug Length (mm)
1: MTA (*n* = 11)	0.569 ± 0.021	2.030 ± 0.133	1.843 ± 0.046	3.900 ± 0.193
2: Biodentine (*n* = 10)	0.557 ± 0.022	2.269 ± 0.174	1.881 ± 0.053	4.015 ± 0.228
3:TotalFill (*n* = 10)	0.578 ± 0.018	2.155 ± 0.075	1.888 ± 0.067	3.481 ± 0.125
4: Guttapercha (*n* = 10)	0.556 ± 0.015	2.171 ± 0.176	1.816 ± 0.059	N/A
5: Intact teeth (*n* = 10)	0.591 ± 0.008	1.780 ± 0.13	2.704 ± 0.098*^,^**	N/A

* *p* < .01 compared to group 1, 2 and 3.

** *p* < .001 compared to group 4; Kruskal-Wallis test with Dunn’s multiple comparison.

### Fracture testing

**Figure 3.**
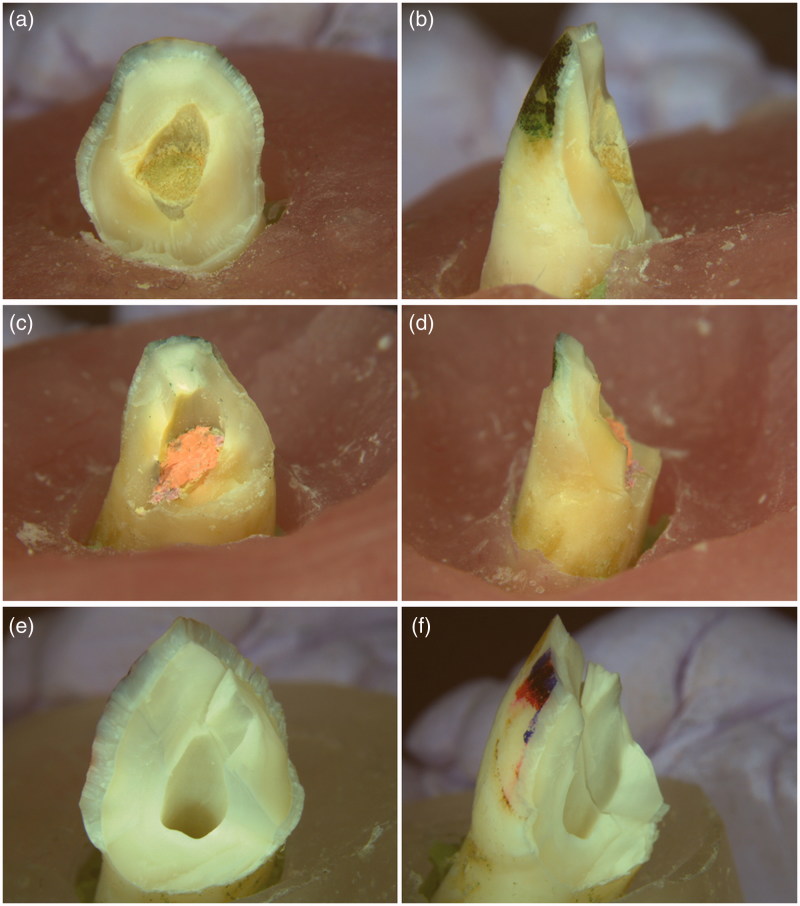
Typical fracture pattern of the immature teeth under the fracture test. (a) and (b): Biodentine group; (c) and (d): Gutta-percha group; (e) and (f): Intact teeth group. The diagonal fracture line extends from the buccal aspect through the canal to the lingual aspect of the tooth. The treated immature teeth fractured at the interface between the material plug/or gutta-percha and composite filling (a-d). The fracture line of the intact teeth is mainly located within the crown (e-f). Lingual aspects: (a), (c) and (e); Lateral aspects: (b), (d) and (f). (×1 Magnification).

All teeth were looked under ×1 magnification for fracture patterns. The diagonal fracture line extends from the buccal aspect of the crown to the lingual aspect of the teeth and exposes the root canal of all tested teeth. The fracture line of the simulated immature teeth (groups 1–4) crosses the interface between the material plug or gutta-percha and composite filling (cervical area) whereas the fracture line of the intact teeth (group 5) is mainly located within the crown (Figure 3).

**Figure 4.**
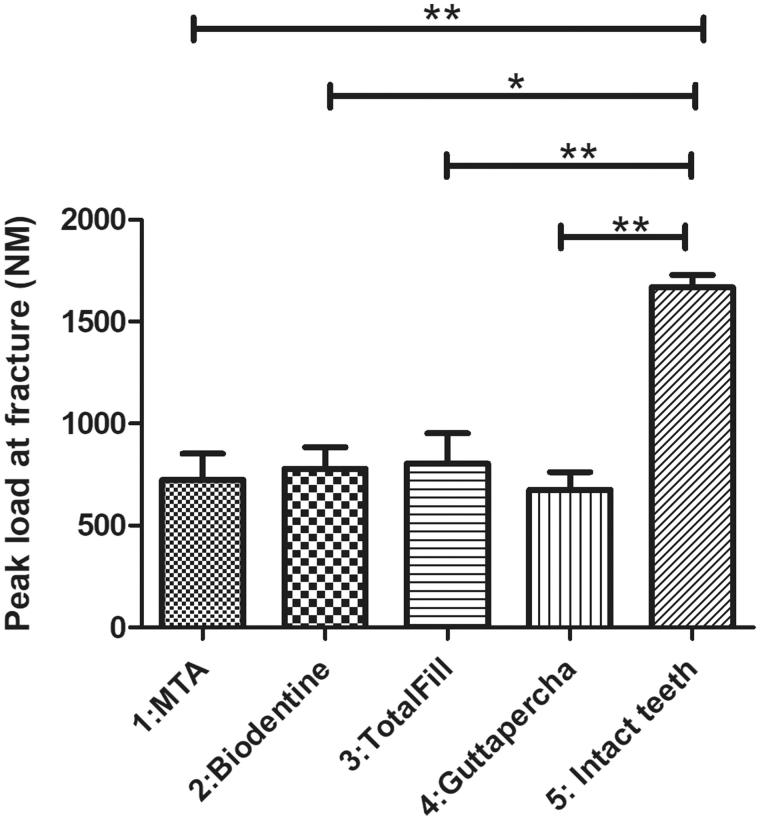
Intact teeth showed a significantly higher peak load to fracture in comparison to the other four groups (1669 ± 60.77 N). Simulated immature teeth filled with gutta-percha showed the lowest peak load to fracture (GP: 675.8 ± 86.84 N). Simulated immature teeth filled with TotalFill showed a higher peak load to fracture (804.5 ± 147.8 N) compared to the other TSCs (MTA: 724.2 ± 128.2 N and Biodentine: 779.4 ± 104.7 N). However, there was no statistically significant difference between the simulated immature teeth regardless of the material. Results are presented as mean ± SEM, Kruskal-Wallis test with Dunn’s multiple comparison, *p < 0.05; **p < 0.01.

The result of fracture testing showed that intact teeth (Group 5) had a significantly higher peak load to fracture (1669 ± 60.77 N) in comparison to all other test groups (Figure 4). TotalFill had a higher peak load to fracture (804.5 ± 147.8 N) in comparison to MTA (724.2 ± 128.2 N) and Biodentine (779.4 ± 104.7 N) whereas the GP control group 4 exhibited the lowest peak load to fracture among all simulated immature teeth (675.8 ± 86.84 N). However, there were no statistical significant differences among the simulated immature teeth (groups 1–4) (Figure 4).

## Discussion

The experiment model in this study emphasizes the immediate effect of the TSCs on treated immature teeth with REPs. We implemented a continuously increasing load of force model to measure fracture resistance. Traumatic dental injuries involve mostly anterior teeth [2]. The absence of high occlusal forces at the incisors may imply that the type of force that leads to dental trauma in such cases is a single impact that overwhelms the structural integrity of the tooth at that moment. Untreated immature bovine teeth had a higher fracture resistance than immature bovine teeth treated with TSCs therefore, the null hypothesis was rejected. Under the experimental set-up, the treated immature teeth fractured at the cervical area and thus, REP and cervical seal with bioceramic materials does not seem to reinforce fracture resistance of bovine teeth.

Bovine teeth were used and prepared to simulate immature teeth. Use of human teeth for the same purpose would have would have allowed for testing the hypothesis in a more clinically relevant substrate. However, difficulty to obtain sufficient quantity and with adequate quality, as well as ethical issues led to use of bovine teeth. All teeth used were extracted from animals of approximately same age shortly after slaughtering and stored under the same conditions until preparation. Thus, minimizing variations in morphology and composition. Previous studies comparing human and bovine teeth showed similar dentin tensile strength and modulus of elasticity [21], fracture strength of composites [22], as well as dentin Knoop hardness [23], properties relevant to the current experimental model. Although human teeth are generally preferred for *in vitro* dental research, bovine teeth were a valid substitute in this study.

We opted to simulate immature roots with a certain root length (15 mm) consistent with stage 3 development [1,24–26]. This length was chosen as shorter roots, typical of earlier root development stages, were easily dislodged from the acrylic mold during loading. Furthermore, the crown was also standardized (9 mm) for all tested teeth and the canals of the prepared ones were enlarged to a canal diameter of approximately 2.2 mm, significantly larger than the canal of the intact teeth (ca 1.8 mm). An earlier report with similar experimental set-up concluded that teeth with a canal diameter of 1.5 mm or less does not need canal wall reinforcement after endodontic treatment [27] and the intact teeth in the current study exhibited a similar lumen diameter. The majority of teeth treated with REPs are teeth in stages 2 through 5 [28] and thus, the current preparation was suitable for the scope of this study.

In addition, the experimental set-up included simulation of the PDL. An elastomeric impression material was used as in previously evaluating ex vivo tooth fracture resistance models [5,29]. The modulus of elasticity of human PDL ranges from 0.12–0.96 MPa [30], which is comparable to various elastomeric impression materials [31] as the thin layer of polyvinylsiloxane used in the current study. Soares et al showed that PDL simulation had a significant effect on fracture resistance in a similar ex vivo laboratory model [29]. The presence of PDL is important when teeth are subjected to trauma. It plays a major role in the stress distribution of forces applied to teeth [32]. For all of the above, the present model is suitable for testing the hypothesis.

Previous studies investigating the intraradicular reinforcement of structurally compromised roots showed that the resistance to fracture was directly related to the remaining tooth structure and to the amount of dentin at the cervical area [33,34]. This was confirmed by the current study as the intact teeth with wide canal but higher dentin thickness at the cervical area (control teeth), required double the force in order to sustain fracture under the experimental set up.

Simulated immature teeth treated with REP showed no difference in fracture resistance compared to teeth filled with gutta-percha in the current study. Group 4 (GP) acted as negative controls and was expected to exhibit lower resistance to fracture compared to the intact teeth. In clinical situation, immature teeth filled with gutta-percha represent cases treated with apexification technique. Apexification with long-term calcium hydroxide treatment has been banned as responsible for cervical fractures due to the effect of calcium hydroxide on dentin structure [1,35]. Therefore, direct apexification techniques with the use of an apical plug of bioceramic materials have been advocated as the preferable method of treatment for necrotic immature teeth. In the current study, the GP group was not filled with an apical plug of TSCs in order to facilitate the obturation of the wide canal. The reason was purely financial, and it would not have an effect on fracture resistance at the cervical area. Moreover, we did not treat the GP group with long-term calcium hydroxide dressing prior to root canal obturation with gutta-percha. All teeth were treated with the same REP protocol and all prepared canals were subjected to the same chemical treatment with irrigation and antibiotic dressing in order to avoid possible structural changes of the dentin. There is evidence that long-term and periodic changes of the intracanal dressing may negatively affect fracture resistance of teeth [19]. It is unlikely that the low resistance to fracture exhibited by the negative controls was due to structural changes of the dentin after the chosen REP protocol since it was a short-term treatment. Nevertheless, all treated and filled immature teeth in this study showed low resistance to fracture regardless of filling material.

The sample size used in this study was sufficient to demonstrate differences between intact and treated teeth. Lack of reinforcement in fracture resistance of simulated immature teeth when bioceramic materials were applied at the cervical area was the main finding of this study. There were small, non-significant differences in fracture resistance between the tested TSCs. However, increased number of teeth would be needed in each group in order to detect possible differences between the tested TSCs as indicated by the current results. It would have been interesting to further investigate if the choice and the thickness of bioceramic material at the cervical area plays a role in fracture resistance at this vulnerable area.

There are several studies trying to address a similar question with conflicting results but the difference from the current study was that the entire immature canal was filled with TSCs [20,36–39]. Within the limitations of *in vitro* studies, canal filling with MTA, or other bioceramic materials e.g. calcium phosphate bone cement, or BioAggregate have been reported to reinforce fracture resistance in some studies [20,36,39,40] whereas in others, the materials used did not [17,37,38]. Most of the studies have used MTA as the golden standard. However, the discoloration caused by MTA even when placed below the CEJ [41] have led to the use of other TSC during REP such as Biodentine or TotalFill. To the best of our knowledge, this is the first study applying these three commonly used TSC at the cervical area only according to advocated REPs and evaluating their effect in fracture resistance *ex vivo*.

The use of composite resin systems has been recommended for the reinforcement of the cervical area of treated immature teeth [16,42,43] and placement of composite restoration is often the final step in the treatment of traumatized immature teeth. The lack of difference in fracture resistance shown under the current experimental set-up between the treated immature teeth (groups 1–4) may also attributed to the composite restoration.

## Conclusions

Within the limitations of this study, we can conclude that TSC such as MTA, TotalFill and Biodentine do not influence either negatively or positively, the fracture resistance of immature teeth during regenerative endodontic therapy. Further material tests and clinical trials are necessary to validate these results.
